# Unibody design for aortic disease with a narrow aortic bifurcation: tips and tricks for success

**DOI:** 10.1590/1677-5449.200230

**Published:** 2021-09-06

**Authors:** Ricardo de Alvarenga Yoshida, Renato Fanchiotti Costa, Débora Ortigosa Cunha, Rafael Mendes Palhares, Rodrigo Gibin Jaldin, Marcone Lima Sobreira, Rafael Elias Farres Pimenta, Winston Bonetti Yoshida

**Affiliations:** 1 Universidade Estadual Paulista “Júlio de Mesquita Filho” – UNESP, Faculdade de Medicina de Botucatu, Botucatu, SP, Brasil.; 2 Sociedade Brasileira de Angiologia e de Cirurgia Vascular – SBACV, São Paulo, SP, Brasil.

**Keywords:** abdominal aorta, aortic diseases, saccular aneurysm, aneurysm, endovascular procedures, aorta abdominal, doenças da aorta, aneurisma sacular, aneurisma, procedimentos endovasculares

## Abstract

**Background:**

Surgical management of patients with abdominal aortic diseases associated with distal narrowing is a challenging situation.

**Objectives:**

To evaluate outcomes of unibody bifurcated endovascular stent graft repair.

**Methods:**

This is a retrospective, observational, multi-institutional database study of a cohort of consecutive cases, approved by the local Ethics Committee. Records were reviewed of patients diagnosed from 2010 to 2020 with “shaggy” aorta, saccular aneurysm, penetrating aortic ulcer, and isolated aortic dissection located in the infrarenal abdominal aorta. All patients were treated with a unibody bifurcated stent graft. Main outcomes were technical success, procedure complications, long-term patency, and mortality in the follow-up period up to 5 years. Data on demographics, comorbidities, surgical management, and outcomes were analyzed.

**Results:**

Twenty-three patients were treated with unibody bifurcated stent graft repair, including 7 cases of “shaggy” aorta, 3 isolated dissections of the abdominal aorta, 4 penetrating aortic ulcers, and 9 saccular aneurysms. Immediate technical success was achieved in 100% of cases. At follow-up, all stent grafts remained patent and there were no limb occlusions. The patients were symptom-free and reported no complications related to the procedure. There were 5 deaths during the follow-up period (median= 4 years), but none were related to the procedure and there were no aorta-related deaths.

**Conclusions:**

The present study shows that unibody bifurcated stent grafting is safe and effective in this group of patients with narrow distal abdominal aorta and complex aortic pathology. The results were similar for both infrarenal aortic aneurysms and aorto-iliac atherosclerotic disease.

## Resumo

**Contexto:**

O manejo cirúrgico de pacientes com doenças da aorta abdominal associadas ao estreitamento distal é desafiador.

**Objetivos:**

Avaliar os desfechos do reparo endovascular com endoprótese bifurcada do tipo monobloco.

**Métodos:**

Foi realizado um estudo retrospectivo, de coorte observacional, de banco de dados multi-institucional, com casos consecutivos, após aprovação pelo Comitê de Ética local. Foram analisados prontuários de pacientes com diagnóstico de *shaggy aorta*, aneurisma sacular, úlcera penetrante da aorta e dissecção isolada da aorta localizados na aorta abdominal infrarrenal entre 2010 e 2020; todos os pacientes foram tratados com endoprótese bifurcada do tipo monobloco. Os principais desfechos foram sucesso técnico, complicações relacionadas ao procedimento, perviedade de longo prazo e mortalidade no seguimento de até 5 anos. Foram incluídos dados demográficos, comorbidades, manejo cirúrgico e desfechos.

**Resultados:**

Vinte e três pacientes foram tratados com endoprótese bifurcada do tipo monobloco, incluindo 7 casos de *shaggy aorta*, 3 dissecções isoladas da aorta abdominal, 4 úlceras penetrantes da aorta e 9 aneurismas saculares. Sucesso técnico imediato foi obtido em 100% dos casos. No seguimento, todas as endopróteses permaneceram pérvias e não houve oclusões de membros. Os pacientes estavam sem sintomas e não relataram complicações relacionadas ao procedimento. Ocorreram 5 óbitos durante o seguimento (mediana = 4 anos), mas nenhum relacionado ao procedimento ou à aorta.

**Conclusões:**

O presente estudo mostra que a endoprótese bifurcada do tipo monobloco é segura e eficaz neste grupo de pacientes com estreitamento distal da aorta abdominal e patologia aórtica complexa. Os resultados foram semelhantes para aneurismas da aorta infrarrenal e doença aterosclerótica aortoilíaca.

## INTRODUCTION

Penetrating aortic ulcer,^1^^,^ 2 saccular aneurysm,^3^^,^ 4 diffuse atherosclerotic aortic disease (“shaggy aorta”),^5^^-^ 7 and isolated dissection of the abdominal aorta^8^ constitute a group of heterogenous and infrequent aortic lesions with a normal or narrow diameter of the abdominal aortic bifurcation.^2^^,^ 9 We have arbitrarily called this disease group “narrow distal abdominal aortic diseases” (NDAAD). Surgical treatment may be performed using conventional open surgery or an endovascular procedure.^10^

Despite some technical limitations, endovascular treatment has become the most widely used treatment option, with the potential to reduce morbidity and mortality associated with these challenging aortic lesions.^10^^-^ 12 In addition, the procedure is less invasive than conventional surgery.^12^^,^ 13 However, even though there is a wide range of stent grafts available on the market, standard bifurcated stent grafts require a diameter of at least 18-22 mm at the aortic bifurcation (distal aorta). Potential risks in patients with a narrower distal aorta include arterial dissection, disruption, thrombosis, difficulties with contralateral leg opening and gate catheterization, or external limb compression, which may cause it to collapse. The unibody bifurcated stent graft is anatomically fixed and stabilized at the aortic bifurcation, both branches are directedly released in the common iliac arteries and the contralateral leg is pre-loaded. These design features eliminate the need for gate catheterization and minimize the problems caused by these anatomical constraints. There are a small number of publications that highlight use of a unibody bifurcated stent graft for treatment of NDAAD. It has predominantly been used in atherosclerotic and non-aneurysmal aortic diseases, but these studies report a small number of patients and none have included all NDAAD together in a single series.^13^^,^ 14 In another study involving 112 patients with abdominal aortic aneurysms with narrow distal aorta, the unibody bifurcated stent graft was only used in 10 cases, with the advantage of not requiring gate catheterization.^12^

The purpose of the present study was to evaluate our experience of the safety and efficacy of endovascular treatment of NDAAD restricted to the infrarenal aorta segment exclusively using unibody bifurcated stent grafts.

## METHODS

This is a retrospective, observational, multi-institutional (2 independent centers) study of a cohort of consecutive cases with “narrow distal abdominal aortic diseases” (NDAAD) treated with an endovascular technique using the Endologix^R^ AFX unibody bifurcated stent graft (Endologix, Inc., Irvine, CA, USA). A narrow aortic bifurcation was defined by outer-to-outer aortic diameter <18 mm measured in any location within the distal 5 cm above the aortic bifurcation.

The study was approved by the Ethics Committee (decision number 3.493.497). The early postoperative period was defined as the 30 days after the operation. Mortality and morbidity were investigated, including arterial or stent graft stenosis, kinking, or occlusion.

Consecutive patients with NDAAD treated with unibody bifurcated stent graft in the period between 2010-2020 were included. Exclusion criteria were treatment with other devices or typical atherosclerotic aortic diseases (occlusive or stenotic disease not characterized as “shaggy aorta”) or NDAAD located in segments of the aorta other than the infrarenal segment. There were no cases with contraindications to use of a unibody stent graft design.

### Procedures

Computed tomography angiography (CTA) was selected as the standard imaging modality for diagnostic confirmation and therapeutic planning in all cases, complemented by digital angiography when necessary. Terarecon^R^ software was used for measuring and planning the procedures. All patients were screened for cardiac risk and those with extensive coronary disease were treated by cardiologists or cardiovascular surgeons before the operation. Nephrology was consulted during the course of treatment of those patients in whom pre-existing renal insufficiency was identified. Spinal anesthesia was performed in 66%, considering surgical risk stratification. Common femoral access was generally obtained by percutaneous puncture under ultrasound guidance (n=15) using the Preclose technique^15^^,^ 16 whenever there were no contraindications or, alternatively, by groin dissection (n=8). The Endologix^R^ AFX stent grafts (Endologix, Inc., Irvine, CA, USA) were implanted using the standard technique described in the manufacturer’s instructions for use, under fluoroscopic guidance with fixed angiographic equipment.

The bifurcated unimodular main body stent graft was introduced via the femoral artery through a 17F sheath and the pre-loaded contralateral leg was captured via the contralateral femoral access with a 9F introducer. The main body measurements and the lengths and diameters of their respective proximal extension devices were chosen according to the measurements of the aorta and the common iliac arteries. In most cases, the main body of the device alone was sufficient for complete NDAAD treatment. Proximal extension devices were required in just five cases to accomplish complete treatment of their main respective diseases. Perclose^R^ Proglide (Abbot Vascular) devices were used for closure of femoral puncture accesses.

Completion angiography with digital subtraction showed good flow through the graft and iliac-femoral arteries. All patients were maintained post-operatively on an antiplatelet regimen of aspirin (100 mg) only, for life.

### Outcomes

The primary outcomes were immediate technical success, procedure complications, patency rates, and short (30 days) and long-term mortality. Secondary outcomes included demographic data, risk factors for atherosclerosis (dyslipidemia, hypertension, current and former smoking, diabetes, coronary artery disease [CAD]), surgical risk factors, cardiac risk, renal function, and presence of clinical symptoms related to the respective conditions. In addition, survival free of symptoms (such as pain, bleeding, infections) and complications related to the procedure (leaks, ruptures, fractures, dislocations, occlusion of branch or stent graft main body) were assessed.

**Figure 1:**
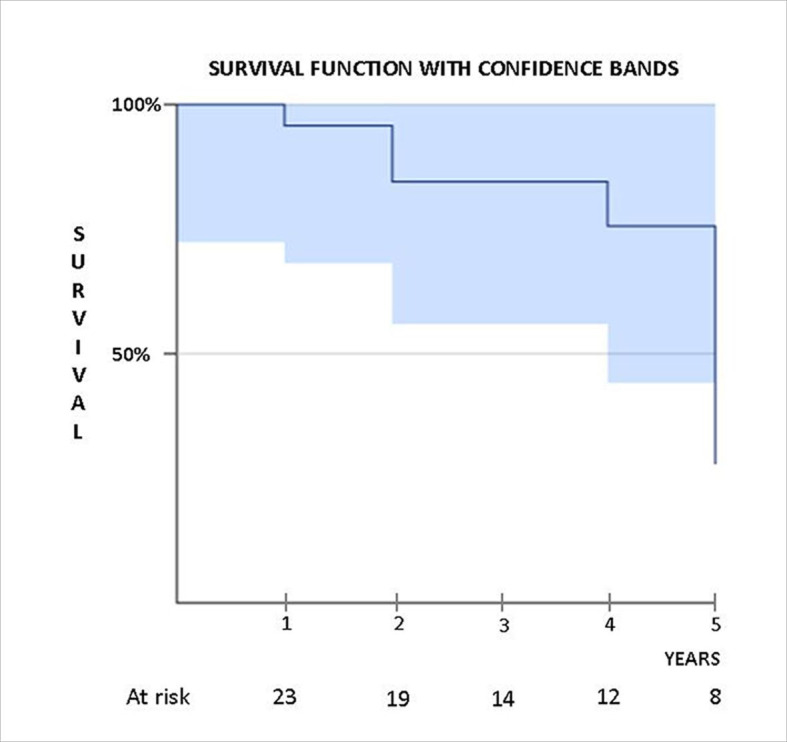
Overall survival rates over time (5 years’ follow-up).

The statistical analysis consisted of descriptive statistics with calculation of proportions. Sample selection was by convenience, attempting to include all cases with the same profile of diagnosis and treatment. The Kaplan Meier survival curve was plotted considering a follow-up period of 5 years and median of 4 years (Figure 1).

## RESULTS

From 2010 to 2020, 23 consecutive NDAAD patients underwent treatment. Demographic data for these patients are shown in Table 1.

**Table 1:** Demographic data and risk factors.

**Demographic Characteristics**	**Shaggy Aorta** **n=7**	**Penetrating Aortic** **Ulcer** **(PAU)** **n=4**	**Saccular Aortic Aneurysm** **(SAC)** **n=9**	**Aortic Dissection** **(DIS)** **n=3**	**%** **n=23**	**P** **T Test and Fisher test**
Age Mean±SD (Range)	72.5±8.97 (59-82)	64.5±5.80 (59-70)	74.3±10.6 (59-90)	64.3±7.23 (56-69)		SAC × PAU p= 0.0292 SAC × DIS p=0.038
Male	5	2	6	2	65%	0.91036
Female	2	2	3	1	34%	
Hypertension	6	3	7	2	78%	0.92068
Diabetes	4	1	4	1	43%	0.749
Renal insufficiency	0	0	2	0	8%	0.20143
Dyslipidemia	6	3	6	2	82%	0.8411
Former smoker	3	1	3	0	30%	0.41903
Smoker	2	3	3	1	39%	0.13147
Coronary artery disease	4	1	2	0	30%	0.17726
Complications	-	-	1 CFA* occlusion, 1 angina + strokes, 1 femoral murmur	-		
Mortality In years after surgery	2 and 5 Unknown causes	-	1 (AMI**), 2 and 7 (pneumonia)	-		

* CFA = common femoral artery;

** AMI = acute myocardial infarction.

Except for one case of traumatic abdominal aortic dissection that required urgent treatment, all patients were evaluated pre-operatively considering cardiac risk, renal function, current medication, introduction or maintenance of antiplatelet therapy, and preparation for surgical procedures. One patient had renal insufficiency (Stage II) and one patient had renal failure (Stage III). All other patients had normal renal function.

The femoral access sites for the Endologix^R^ AFX stent graft (17F) main body were obtained by femoral exposure in 7 cases and by percutaneous access in 16 cases. Percutaneous access was obtained by femoral artery puncture under vascular ultrasound guidance using the Preclose technique,^15^^,^ 16 respecting its indications and contraindications. In all contralateral accesses (9F), the femoral artery was punctured and hemostasis was achieved with a sealing device (Perclose^R^). One patient developed a small pseudoaneurysm as a complication of percutaneous femoral access for main body device implantation (17F), which was successfully treated with ultrasound-guided compression.

The following cases are examples of each type of aortic disease.

### Shaggy aorta (diffuse atherosclerotic aortic disease)

**Figure 2:**
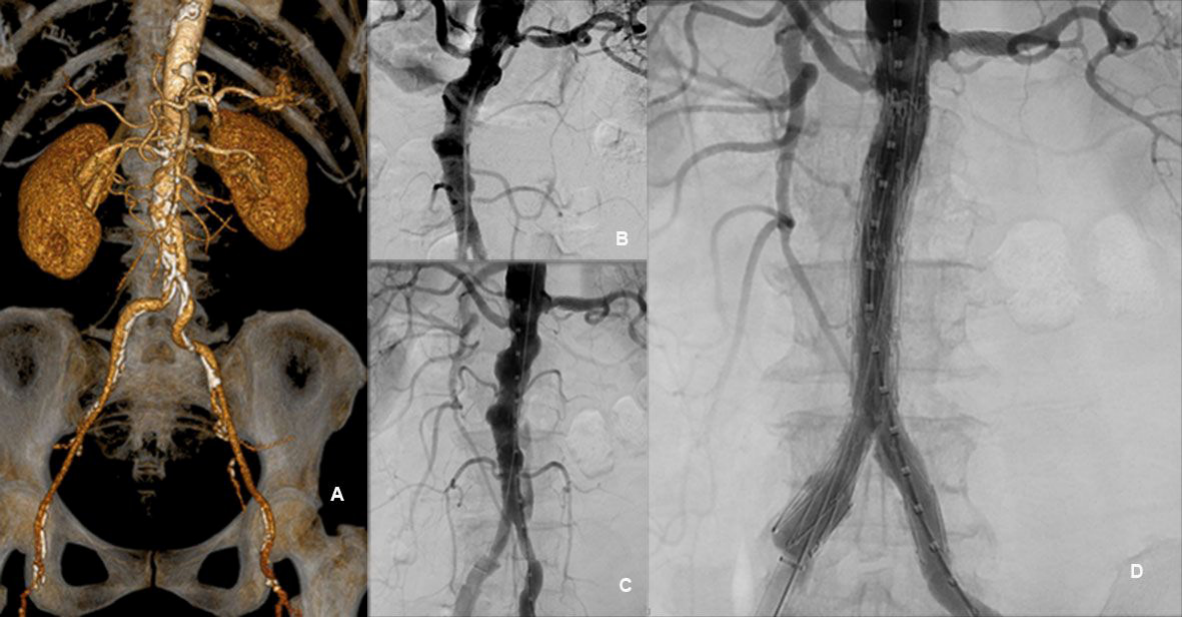
Example of “shaggy” aorta treated with main unibody bifurcated stent graft and proximal extension devices. (A) Pre-treatment Angio-CT image; (B) Intraoperative angiography; (C) Angiography after left renal artery stenting; (D) Post-treatment angiographic appearance.

Seven cases of “shaggy Aorta” were treated. Surgical intervention was performed due to symptoms of severe claudication (Rutherford 3), associated with aortoiliac occlusive disease in 4 patients, while 3 patients had symptoms due to distal embolization (blue toe syndrome, see Figure 2). Distal embolization during the procedure were avoided by using hydrophilic guidewires and soft maneuvers during navigation of devices. No distal embolization occurred during these procedures. Mortality rates are shown in Table 1.

### Saccular Aortic Aneurysm (SAA)

**Figure 3:**
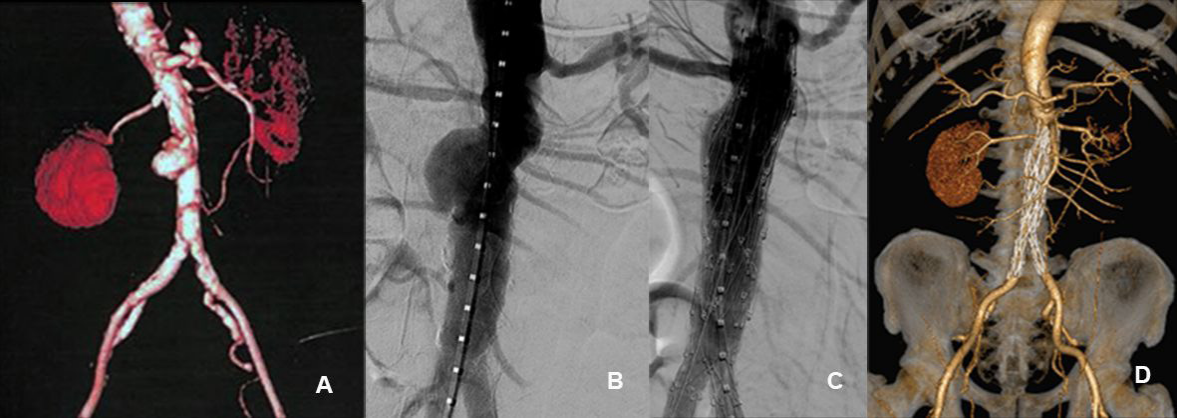
Example of saccular aortic aneurysm treated with main unibody bifurcated stent graft and proximal extension devices. (A) Pre-treatment Angio-CT image; (B) Intraoperative angiography; (C) Post-treatment angiographic appearance; (D) Post-treatment control Angio-CT.

There were nine cases of abdominal aortic saccular aneurysm. Average aortic dilation was 4.3 cm. Main complications and mortality rates are shown in Table 1. All stent grafts remained patent, with no leaks during the follow-up period. Follow-up of the remaining patients was free from symptoms and there were no complications (Figure 3).

### Penetrating Aortic Ulcer (PAU)

**Figure 4:**
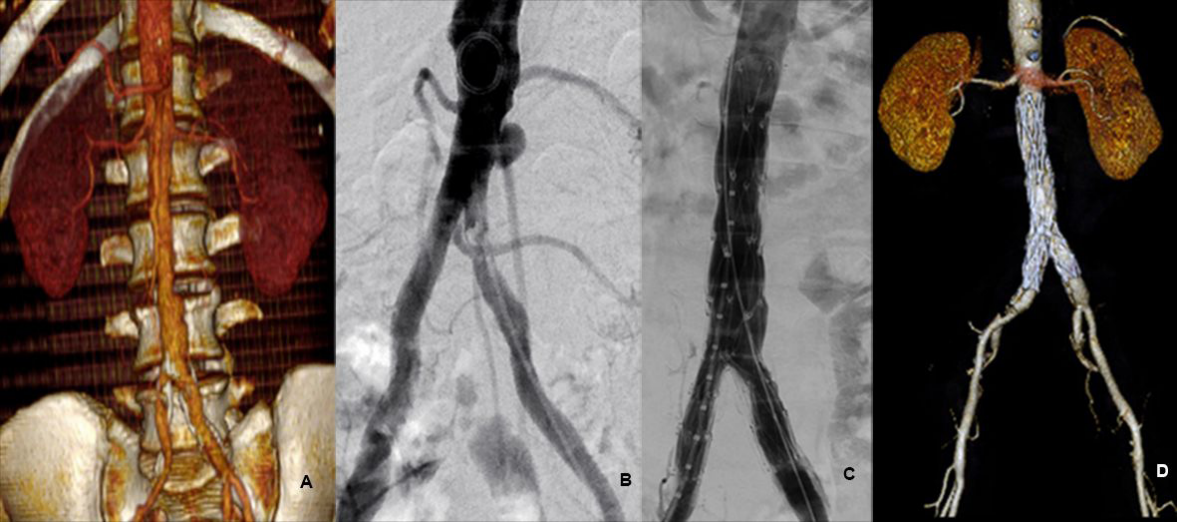
Example of PAU treated with main unibody bifurcated stent graft device only. (A) Pre-treatment CTA image; (B) Intraoperative angiography; (C) Post-treatment angiographic appearance; (D) Post-treatment control CTA.

There were 4 cases of symptomatic PAU (abdominal pain). Although the indications for surgery of abdominal aorta PAU are not well established, symptomatic disease is the main indication for surgery due to elevated risk of rupture (20-50%).^1^^,^ 17 ^,^ 18 After surgery, the patients remained free from symptoms or complications during the follow-up period. (Figure 4).

### Isolated abdominal aortic dissection

**Figure 5:**
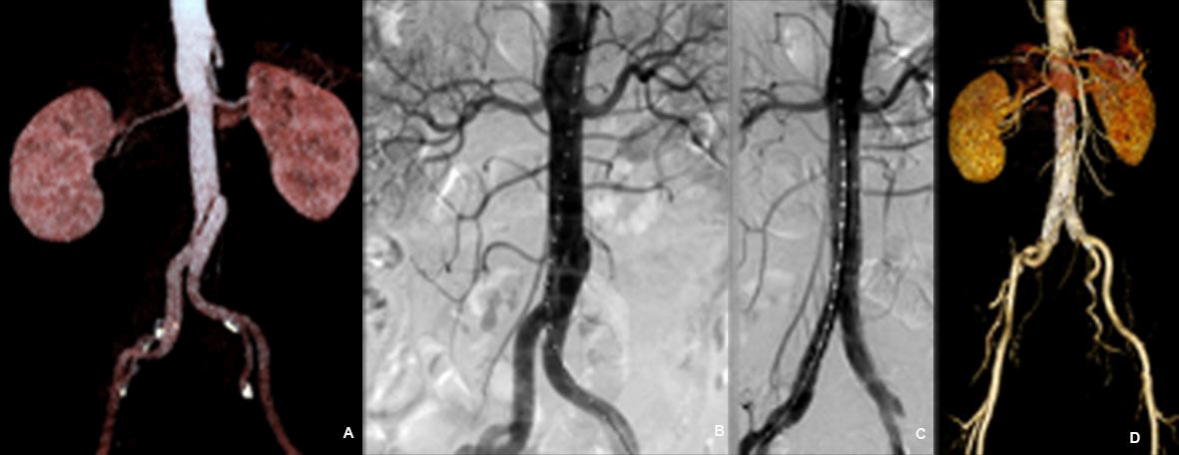
Example of isolated abdominal aortic dissection treated with main unibody bifurcated stent graft device only. (A) Pre-treatment CTA image; (B) Intraoperative angiography; (C) Post-treatment angiographic appearance; (D) Post-treatment control CTA.

There were 3 cases of isolated abdominal aorta dissection. Indications for surgery were as follows: one patient already had aneurysmal degeneration, another patient with risk factors for atherosclerosis had spontaneous dissection (diagnosed as an incidental finding during investigation of atypical abdominal pain), and a third patient presented with dissection caused by an abdominal trauma against a concrete structure after a fall from a height of 3 meters, with severe abdominal pain unamenable to analgesic medications (intractable pain) and underwent urgent surgery. Angio-CT and intraoperative angiography provided all information needed for treatment and IVUS was not needed. All patients remain in clinical follow-up with no symptoms or complications (Figure 5).

Blood loss was negligible in all procedures and no transfusions were needed. All patients spent at least 12 hours in the intensive care unit (ICU) and no patients in this cohort remained in the ICU longer than 24 hours. Renal function was maintained at baseline pre-operative values. The mean length of hospital stay was 3.6 days.

Post-procedure follow-up clinical assessments were performed at 3 months, 6 months, 1 year, and annually thereafter. Figure 1 shows the Kaplan-Meyer survival curve (bounded by the error interval) over the 5-year follow-up period. Follow-up imaging examinations were conducted at 6 months, 1 year, and thereafter by CTA or vascular Doppler ultrasonography. Only one patient was lost to follow-up. There were no limb occlusions or stent graft complications.

## DISCUSSION

Successful treatment of a narrow distal aortic bifurcation with standard endovascular devices can be challenging We report our experience with treating this anatomy with a unibody design. In general, the demographic profile was: predominantly older, male, hypertensive, and dyslipidemic patients, compatible with a pattern of atherosclerosis, aneurysm, and arterial dissection risk factors. About 40% were diabetic and around 1/3 of the patients were smokers and had coronary disease, which are also risk factors for peripheral atherosclerosis.

Cases of diffuse atherosclerotic aortic disease (“shaggy aorta”) associated with lower limb ischemia (symptomatic PAD) or distal embolization of atheromatous plaque fragments (blue toe syndrome) should be treated surgically.^19^ In such patients, the conventional surgery options are an aorto-bifemoral or axillary-bifemoral bypass with ligation of the iliac arteries in cases with distal embolization.^20^ However, high morbidity and mortality rates, mainly due to difficulties in aortic clamping, anastomoses of calcified arteries, and presence of aortic mural thrombi are the main limitations of these techniques. On the other hand, the unibody bifurcated stent graft was able to completely cover all the inner surface of the aorta and iliac arteries, thus preventing any embolization of fragments to peripheral distal arteries, as well as preserving the native anatomy of the affected vessels. Conversely, the use of uncovered aortic stents would not avoid passage of micro fragments through the mesh. In addition, due to excessive calcification, accidental rupture of the aorta or iliac arteries during dilation (as in angioplasty), they would not cover and prevent a hemorrhagic complication.

PAU in the abdominal aorta occurs in only 17% of cases^21^^,^ 22 and requires specific treatment, mainly in symptomatic cases. Accurate diagnosis is very important to define the best therapeutic approach.^23^ The conventional surgery option is an aorto-aortic bypass. In contrast, endovascular treatment is a less invasive alternative that is widely performed (70% of cases) with low complications rates (0-11%).^24^^-^ 26 Literature reviews show that PAU is associated with high risk of rupture (20-50%) and mortality (80%) and urgent surgical treatment is recommended.^1^^,^ 17 ^,^ 18

For isolated acute abdominal aorta dissections with refractory abdominal pain and limb ischemia or for chronic aneurysm degeneration, endovascular treatment is associated with lower mortality rates and perioperative complications. This treatment avoids both aortic clamping and sutures in the highly friable aortic wall.^27^ In addition, it allows coverage of the false lumen entry site and false lumen compression and effectively favors its thrombosis with the true lumen expanded.^27^^-^ 29

Saccular aneurysm of the abdominal aorta is associated with higher risk of rupture compared to the typical fusiform configuration; extreme thinning and weakening of the aortic wall is associated with asymmetric dilation or focal protuberance.^4^ Although the recommended treatment is not clear in the literature, most guidelines suggest saccular abdominal aortic aneurysms should be repaired.^30^ With the technical advancement of endovascular procedures, this surgery can be performed with considerably lower morbidity and mortality (2-4%) than conventional surgery and with better results.^31^

Narrow diameter at the aortic bifurcation has been recognized as a risk factor for stent graft limb collapse and occlusion and arterial disruption.^12^ Several conventional or endovascular techniques may usually be employed to treat NDAAD. In the case of the endovascular technique, careful evaluation of the anatomy, location and extent of the lesion and selection of the appropriate device yield good results. Many types of bifurcated endografts (bi/tri-modular or unibody) can be employed to treat NDAAD^32^ and each of them has technical and structural characteristics that are more appropriate to certain anatomical profiles and particular patient conditions.^33^ Other endovascular devices may also be used, such as Multilayer stents,^34^ parallel covered stents,^35^ or the endovascular sealing technique (EVAS),^36^ but consensus on their use requires further studies.^37^^,^ 38

The diameters of most other bifurcated devices when fully expanded range from 20 to 26 mm, which may cause collapse of the contralateral gate and prevent successful catheterization with the standard technique.^12^ Many maneuvers could be used, such as catheterization up and over the flow divider and pre-dilation of the contralateral gate, but during these maneuvers, proximal and distal migration of the main body stent graft could occur and may cause accidental coverage of renal arteries or collapse.^12^ Other strategies to deal with the limitations of standard bi or tri-modular stent grafts in narrow aortas include forced dilatation of the aortic bifurcation with noncompliant kissing balloon angioplasty and use of kissing stents with balloon-expandable stents after recoil of the angioplasty, especially in very narrow aortic diameters or when severe calcification is present.^12^

The authors considered the AFX device safer and effective to treat this particular group of NDAAD due to its unibody bifurcated stent graft configuration, with pre-loaded contralateral leg, no need of an appropriate minimum diameter in the distal aorta for opening and catheterization of the contralateral leg, and anatomical fixation enabling a safe “paving and cracking” maneuver without additional procedures, if necessary. These characteristics of the AFX device could avoid critical maneuvers which are frequently needed in standard bi-modular or tri-modular stent grafts, and may decrease the morbidity of the procedure. Another advantage of the AFX - Endologix^R^ stent grafts is its profile (17F for main body device and 9F for contralateral access), allowing percutaneous treatment using the Preclose^R^ technique. However, the device requires common iliac arteries with more than 30 mm length, and appropriate aortic and iliac artery diameters and length, due to the limited range of diameters and lengths available.

In cases of abdominal aortic aneurysms, especially with exaggerated angles and/or short necks, an increased frequency of proximal extension disconnection from the main body has been described, which has limited the indications for the AFX^R^ endograft in these cases and provoked changes to its design.^39^ This is not a concern in NDAAD because the stent graft is well-fitted inside the aorta, which, as a rule, has no tortuosity in this condition (NDAAD). Moreover, proximal extensions are usually sufficient for the complete treatment of these lesions, without leaks or disconnections.

Long-term studies with the AFX - Endologix^R^ stent grafts in other “off label” uses in occlusive aortic diseases showed a primary patency rate of 78.8% at 36 months (n = 91 cases), with an assisted patency rate of 97.4% over the same period.^13^ In the present study, we observed similar rates for local complications (Table 1) and long-term patency (Figure 1).

Due to the low frequency of NDAAD, limitations of this study were the small and heterogenous sample (which precludes more effective conclusions and generalizations), the fact that this is a retrospective review, and absence of a control group. Since NDAAD are mostly infrequent conditions, a possible selection bias may occur due to the insufficient sample size. Nevertheless, our patients were consecutive, presented similar risks, and a single type of stent graft was used.

Most previous studies of use of unibody stent grafts were anecdotal case reports or series of a few cases of atherosclerotic disease treatment.^13^^,^ 14 Only one article showed results of the use of bifurcated aortic stent grafts in AAA with narrow aortas, but as part of a larger series (n=112), with only 10 cases of this particular stent graft.^12^ Another study presented lithotripsy as an adjuvant technique for endovascular repair of complex saccular aortic aneurysm with narrowed aorta.^40^ The present study gives additional support for “off label” use of unibody bifurcated stent grafts in NDAAD and emphasizes that critical maneuvers to prevent limb graft occlusion or accessing the contralateral gate using up and over technique or brachial approach are generally unnecessary with a unibody stent graft,^12^ which may be reflected in lower morbidity.

## CONCLUSIONS

The present series showed potential appropriate applications and favorable results of AFX-Endologix^R^ unibody bifurcated stent graft in this group of NDAAD suggesting that it was an effective technique with patency rates similar to those of endovascular treatments of abdominal aortic aneurysms and aorto-iliac atherosclerotic disease,^13^^,^ 33 with no need for critical associated maneuvers.^12^ There were low complication rates and no associated mortality. Patients with this group of diseases may benefit from this endovascular treatment and, considering that there are other device options, further studies with larger numbers of patients are necessary for more accurate conclusions.
